# Secondary School Students’ LLL Competencies, and Their Relation with Classroom Structure and Achievement

**DOI:** 10.3389/fpsyg.2016.00680

**Published:** 2016-05-09

**Authors:** Julia Klug, Marko Lüftenegger, Evelyn Bergsmann, Christiane Spiel, Barbara Schober

**Affiliations:** Department of Applied Psychology: Work, Education and Economy, University of ViennaVienna, Austria

**Keywords:** lifelong learning, self-regulated learning, motivation, classroom structure, achievement, achievement goal, secondary school

## Abstract

There is a strong urge to foster lifelong learning (LLL) competencies with its key components – motivation and self-regulated learning – from early on in the education system. School in general is presently not considered to be successful in systematically imparting motivation and self-regulated learning strategies. There is strong evidence that decisive motivational determinants decrease the longer students stay in school. At present, the central sources of information about the situation in Austria are international monitoring studies, which only examine selected aspects of specific target groups, and their interpretability concerning mean values is constricted due to cultural differences. Thus, it is important to conduct additional and more differentiated national surveys of the actual state. This is why this study aimed at answering the following questions: (1) how well are Austrian students equipped for the future, in terms of their lifelong learning competencies, (2) can perceived classroom structure predict students’ LLL, and (3) is there a correlation of students’ LLL with their achievement in the school subjects math and German language. 5366 students (52.1% female) from 36 Austrian schools took part in the online-questionnaire (mean age 15.35 years, *SD* = 2.45), which measured their perceived LLL competencies in the subjects math and German language, their perceived classroom structure and their achievement. Results showed that the great majority of Austrian students – independent from domain and sex – know and are able to apply cognitive as well as metacognitive learning strategies. With regard to motivation the picture is less satisfactory: whilst students’ self-efficacy is not the problem, there is a lack of interest in the school subjects and they often report to follow performance approach goals. Classroom structure positively predicted students’ goals, interest, self-efficacy and learning strategies. Self-efficacy, performance approach goals, meta-cognitive and deep learning strategies in turn predicted achievement positively, and performance avoidance goals negatively.

## Introduction

This study aimed at gaining insight into Austrian students’ lifelong learning (LLL) competencies. What are they, and why are they of importance? In a work environment characterized by increasingly rapid change, a higher degree of flexibility and motivation to learn will be demanded of future employees. In recent decades, the European Union has rapidly begun shifting from an industrial to a knowledge-based economy. Therefore, at the beginning of the millennium, the European Commission launched an official strategy for fostering lifelong learning (Commission of the European Communities, 2000). In their early days, discussions on lifelong learning were predominantly focused on the business context (Cendon et al., 2007). However, there is also a strong push to start fostering lifelong learning competencies earlier on in the education system (Zimmerman and Schunk, 1989). Large-scale assessment studies like TIMSS and PISA show that students still have deficits with regard to lifelong learning competencies, which could pose a problem for the future labor market (Organisation for Economic Cooperation and Development [OECD], 2004). Thus, it is strategically critical to promote lifelong learning in the school setting and raise teachers’ awareness of the benefits of these competencies (Wayne and Youngs, 2003; Lipowsky, 2006).

### Lifelong Learning

What are the key components of lifelong learning? Motivation (or the will to learn) and strategies for turning this drive into action are the two key factors for learning (Weinstein and Hume, 1998; Artelt et al., 2003; Schober et al., 2013). With this research project, we wanted to answer the following questions: how well are students in Austria prepared for LLL in terms of motivation and self-regulated learning strategies in two different subjects? Does the perceived classroom structure, meaning what the teacher does in the classroom in terms of promoting autonomy, designing tasks and giving feedback, have an impact on students’ LLL? And finally, how relevant are LLL competencies for school achievement measured by grades in the last school report and a mathematical and German language achievement test?

#### Definition of LLL

The most comprehensive definitions of LLL are presented by the European Commission (2001, p. 9): lifelong learning is “all learning activities, undertaken throughout life, with the aim of improving knowledge, skills, and competence within a personal, civic, social, and/or employment-related perspective.” Defined as so, LLL is not a new concept, but it does enhance our theoretical perspective by adding a lifespan approach to learning. In light of the diverse body of literature and the fact that there is not yet a psychological theory built around LLL, it is crucial to narrow the research focus to two central components: motivation (e.g., Heckhausen and Gollwitzer, 1987; Wigfield and Eccles, 2000) and self-regulated learning strategies (e.g., Zimmerman, 2000; Schmitz and Wiese, 2006). In layman’s terms, the former could be described as appreciation for learning, and the latter would be associated with effective knowledge management. The current project sought to link these concepts of motivation and self-regulated learning strategies with models acknowledging contextual factors such as classroom structure (Ames, 1992; Helmke, 2010; Bergsmann et al., 2013) and achievement.

#### Empirical Findings Concerning LLL

In general, schools are not presently considered to be successful in systematically imparting the aforementioned LLL core components of motivation and self-regulated learning strategies (e.g., Randi and Corno, 2000; Gottfried et al., 2001; Artelt et al., 2003). A large number of international studies have examined various determinants of students’ learning motivation, such as interest, learning goal orientation, and self-efficacy, as well as aspects of self-regulated learning (for an overview, see, e.g., Schunk et al., 2008). Although their results differ in the details, there is strong evidence that key motivational determinants of LLL decrease the longer students stay in school, especially after the transition to secondary school (Wigfield et al., 2006; Fischer and Rustemeyer, 2007; Schunk et al., 2008; Lüftenegger et al., 2012). In some studies, a decline in students’ self-regulated learning behavior has also been reported (e.g., Peetsma et al., 2005). Summing up the findings—including prominent international studies (Organisation for Economic Cooperation and Development [OECD], 2008)—it becomes clear that educational systems and practices in many countries are quite poorly prepared, and often ineffective, when developing LLL competencies in schools.

In light of all this, it is necessary to consider the school context in examining LLL competencies. One contextual factor that has been shown to interact with individual LLL competencies is teaching quality (for an overview, see Van de Grift, 2007). Teaching quality is a heterogeneous field where a great number of concepts and approaches are thrown around, and various dimensions are focused on (Emmer and Stough, 2001; Wayne and Youngs, 2003; Cartel et al., 2006; Clotfelter et al., 2006; Van de Grift, 2007). Amongst other variables that affect students’ learning, one prominent concept in the research on teaching quality is classroom structure (Epstein, 1988, 1989; Ames, 1992). Classroom structure describes how teachers design tasks, the autonomy structure in the classroom, and student achievement evaluation. In a lot of studies a positive effect of classroom structure on various student functioning variables has been shown, some of them being well-being and achievement (for an overview see, Urdan and Turner, 2005). Bergsmann E.M. et al. (2013), for example, showed that a supportive classroom structure was associated with less verbal aggression amongst students later on. The classroom structure provided by the teacher has also been shown to interact with individual motivational aspects (e.g., learning goal orientation, Lüftenegger et al., 2012, 2014) and individual learning strategies (Wolters, 2004). In contrast to these findings, teachers themselves consider their influence in fostering students’ LLL to be low (Spiel et al., 2011). However, so far there are no studies that systematically examine the interrelationships among the classroom structure provided by the teacher and students’ LLL competencies. That is why we chose to investigate this relation in our study.

In addition to empirical findings on contextual factors supporting the development of LLL competencies, information on the effects of students having greater LLL is also relevant. One of the most important outcome variables for schools is student achievement. Students’ achievement levels are the result of multiple factors (for an overview, see, Hattie, 2009). However, in line with our focus on LLL, we concentrate on motivation and self-regulated learning as antecedents. Individual motivational aspects and determinants of self-regulated learning have been shown to be positively associated with achievement (for an overview, see, Schunk et al., 2008; Dresel and Lämmle, 2011). Motivation predicts achievement even if intelligence and previous achievement are controlled for (e.g., Steinmayr et al., 2011). Although self-regulated learning is considered as relevant for success in learning (e.g., Artelt et al., 2001; Streblow and Schiefele, 2006; Dignath and Büttner, 2008; Dignath et al., 2008), heterogeneous and contradictory correlations, especially with achievement, ranging from zero to medium have been found (e.g., Zimmerman, 2000). However, there are no findings concerning the association between Austrian students’ LLL competencies and their achievement as of yet.

#### The Situation in Austria

At present, the central sources of information about the situation in Austria are international monitoring studies (e.g., TIMSS, PIRLS), particularly PISA (Artelt et al., 2003; Haider and Reiter, 2004; Organisation for Economic Cooperation and Development [OECD], 2004; Schreiner, 2007). Depending on the content area under investigation, survey date and age of the students examined, OECD studies come to different conclusions concerning motivation and self-regulated learning. This is not only true of samples from the entire OECD but also with regard to the current situation among Austrian students.

Concerning motivational aspects, the findings of PISA 2003 (Organisation for Economic Cooperation and Development [OECD], 2007) show that Austrian students (similarly to German and Swiss students) report high levels of discipline and self-efficacy in mathematics, as well as low levels of test anxiety in mathematics. In contrast, the findings of PISA 2006 indicate a more negative situation (Schreiner, 2007): compared to the entire OECD sample, Austrian students show an average level of general interest in science. However, their appreciation for science and instrumental and future-oriented motivation to learn science are very low compared to the international sample.

The determinants of self-regulated learning investigated in PISA 2000 (control strategies, effort, persistence, elaboration, and memorization) have been shown to be relatively high in Austrian students. Nevertheless, the correlations with achievement (in this case reading performance) are rather small (*r* = 0.12–0.18; see, Svecnik and Reiter, 2002). The small size of these correlations is not in line with findings reported in the literature about the critical impact of the determinants of self-regulated learning on achievement (Artelt, 1999). Hence, a replication of these findings—with different instruments—would be desirable before using them to make any generalizations.

In summary, large-scale international assessments mostly examine (or more accurately: can only examine) selected aspects of specific target groups. Furthermore, their interpretability with regard to mean values is constrained by cultural differences (Schütte et al., 2007). Thus, it is important to conduct additional, more differentiated national surveys in order to get a better picture of the current situation in Austria.

### The Present Study

Based on the described research gaps, the following research questions were specified for the present study: (1) To what degree do Austrian students possess LLL competencies? (2) Can perceived classroom structure predict Austrian students’ LLL competencies? (3) Can Austrian students’ LLL competencies predict their achievement?

## Materials and Methods

### Sample

In sum, 5366 students (52.1% female) with a mean age of 15.35 years (*SD* = 2.45, Minimum = 9 years, Maximum = 21 years) from 36 schools participated. The students were at the following levels of education: 5th grade (*n* = 288), 6th grade (*n* = 344), 7th grade (*n* = 401), 8th grade (*n* = 479), 9th grade (*n* = 909), 10th grade (*n* = 1126), 11th grade (*n* = 689), 12th grade (*n* = 649), and 13th grade (*n* = 350). 2670 students (50.2%) worked on the math version and 2696 students (49.8%) on the German language version of the questionnaire. The sample was Austria-wide and comprised students from all common school types at the secondary level – including vocational training schools with technical and other core areas.

### Procedure

The present study was conducted in compliance with ethical standards provided by the Austrian Federal Ministry of Health (Bundesministerium für Gesundheit, 1995) and the American Psychological Association [APA] (2010). Accordingly, prior to participation, teachers were informed about the goals of the research, duration, procedure and anonymity of the data. Participation was voluntary at any time, and informed consent was provided. Data was collected and analyzed anonymously.

The Ministry of Education, Arts, and Culture, the municipality’s local school authority, and the school principals all gave informed consent for the survey.

All Austrian secondary schools were invited to participate in the study. The 36 participating schools were the ones whose directory responded voluntarily. The management of each school gave informed consent to the survey. School administrators informed parents and students about the survey, the voluntary nature of participation, and the confidential use of the data. Less than 1% of the students either refused to participate or were not permitted to participate by their parents. The children did not receive compensation for their participation in the study. Whilst trained research assistants administered the survey, the teachers were present. All students who were included in the study completed the online questionnaire during a regular class period (50 min). Data collection took place in school’s PC-room. The items were presented in an online questionnaire. Every participant got admission to the questionnaire with a code. Codes were handed out to students at random. Even numbers were linked to special questions about the school subject Math, uneven numbers were linked to special questions about the school subject German language. Those two subjects were chosen because they are central and potentially different ones in the Austrian school system in order to being able to check whether there are differences in students’ LLL according to school subject.

### Measures

The self-report online questionnaire for students comprised items with regard to both included subjects (Math and German language) concerning LLL competencies (motivation and self-regulated learning strategies), classroom structure, achievement, and socio-demographic information.

All item scores – despite the achievement measures and self-regulated learning strategies – ranged from 1 (disagree) to 4 (agree) whereby a higher score represents a more positive value for the quality in question. Every item was programmed as forced choice, except the items for the achievement tests.

#### Motivation

Motivation as one of the components of LLL was measured referring to two well-known motivational theories: one being the achievement goal theory (Elliot and Harackiewicz, 1996), the other one being the expectancy-value theory (Wigfield and Eccles, 2000).

##### Achievement goals

Following the trichotomous achievement goal conceptualization (Elliot and Harackiewicz, 1996), we assessed the three constructs mastery goals, performance-approach goals, and performance-avoidance goals. The items were developed within the framework of a long-term cooperation with 16- to 18-years-old students. The main target of the cooperation was to devise ecologically valid items by incorporating the language of the target population (Spiel et al., 2013). The validity of the mastery goal scale had already been demonstrated in an empirical study about classroom structure (Bergsmann et al., 2013). The students’ self-report scale consists of a short introduction (“We want to find out what is most important to you about studying for math class. Please mark how well the following statements apply to you. In math/German, I mainly study …”), followed by six items for mastery goals (sample item: “… because I would like to know how to solve the problems”; α = 0.88), four items for performance-approach goals (sample item: “so I will have good grades compared to other students.”; α = 0.88) and four items for performance-avoidance goals (sample item: “so I won’t be one of the less competent students.”; α = 0.89).

##### Interest

Interest in instruction was considered as an indicator of the value component in the expectancy-value theory, and was assessed with three items (based on Kunter et al., 2002). In item formulation, both value and emotional valence (Krapp, 2002) were considered (sample item: “What I learn in math is important to me”; α = 0.74).

##### Self-efficacy

The self-efficacy scale included three items in accordance with Jerusalem and Satow (1999) and was considered as an indicator of the expectancy component in the expectancy-value theory. The scale focuses on students’ efficacy expectations in the classroom (sample item: “I am convinced that I can do well on class assignments and tests in math”; α = 0.74).

#### Self-Regulated Learning Strategies

Following the general cognitive model of learning and information processing (Weinstein and Mayer, 1986) we assessed cognitive and meta-cognitive learning strategies. The use of cognitive strategies for the processing of information was measured with the two subscales surface strategies (*k* = 3; sample item: “I repeat the material until I know it by heart”; “I remember the most important things by reading through them over and over”; “I remember the most important things by reciting them over and over”) and complex strategies (*k* = 3; sample item: “I explain the material to myself in my own words”; “I summarize the most important things”; “I try to really understand the material”).

The scale meta-cognitive learning strategies comprises aspects of planning, monitoring, regulating, and reflection (*k* = 5; “I organize my time sensibly”; “While I am studying, I consider whether I am taking the right approach”; “If I notice that I am not making any progress, I change my learning technique”; “If I find something to be difficult, then I look at it more closely”; “At the end, I check to make sure that I accomplished what I had set out to do,” α = 0.71). The scale consists of a short introduction (“Think about how you usually prepare yourself for a math test. How often do you do the following?”), and of four items (e.g., “Before I start to study, I come up with a good plan of attack.”). Thereby, each item represents another self-regulated learning phase (planning, monitoring, regulation, reflection). The item scores range from 1 (never) to 4 (often) whereby a higher score represents more frequent use of metacognitive learning strategies (Cronbach’s α = 0.70).

#### Classroom Structure

Classroom structure was measured with the three subscales – task, autonomy, and evaluation/recognition (based on Lüftenegger et al., 2014). The task subscale comprised six items (sample item: “In math class, we should set our own learning pace”; α = 0.79), and the autonomy subscale eight items (sample item: “In math class, the students make important decisions together with the teacher”; α = 0.80). Evaluation/recognition consisted of four items (example item: “In math class, we know how to improve based on the feedback we get”; α = 0.65).

#### Academic Achievement

##### School report grade

Academic achievement in mathematics and German language was measured by asking “What grade were you given on your last school report card in math/German?” In Austria, school grades range from 1 (excellent) to 5 (insufficient/fail). For a more comprehensible interpretation of the results, the values were recoded; therefore, in this study, the higher the grade, the higher the academic achievement.

##### Achievement tests

Students’ achievement in mathematics was assessed with the inductive reasoning subscale “numeric series” for mathematics and with the verbal comprehension subscale “finding similarities” for German language. Both subscales stem from the *Prüfsystem für Schul- und Bildungsberatung für 4. bis 6. Klassen, Revidierte Fassung* (Testing System for Scholastic and Educational Counseling, Grades 4–6 – revised version, PSB-R 4-6; Lukesch et al., 2002) and the *Prüfsystem für Schul- und Bildungsberatung für 6. bis 13. Klassen, Revidierte Fassung* (Testing System for Scholastic and Educational Counseling, Grades 6–13 – revised version; PSB-R 6-13; Lukesch et al., 2004). The test assesses intelligence in accordance with Thurstone’s primary factors. In the subtest “Numeric Series,” fifteen series of nine numbers are to be completed. The numbers that contradict the principle upon which the series is based are to be crossed out. In the subtest “finding similarities,” 25 tasks including five words each are to be completed. In every set of words one word that does not match with the other four has to be flagged.

### Data Preparation and Analytical Approach

With a study of this size (*n* = 5366), it is necessary to develop an appropriate strategy to deal with missing values. The common practices of pairwise deletion, listwise deletion, or mean imputation can result in significant reductions in the validity of the results and thus no longer presents a satisfactory option for dealing with missing values (e.g., Schafer and Graham, 2002). Procedures which incorporate the (multiple) imputation of missing values (Schafer and Graham, 2002) lead to more valid results. The rate of individuals omitting items (non-response) in this study was between 3.1 and 12.7% for all items and, as such, small. We accounted for selective attrition with multiple imputations using the mice package in R (van Buuren and Groothuis-Oudshoorn, 2011). As the LLL determinants and classroom structure are measured as ordinal-level variables, a robust weighted least squares estimator (WLSMV; Muthén, 1984) was used in all analyses in the statistical software package Mplus 7.31 (Muthén and Muthén, 1998–2015).

In a first step, confirmatory factor analyses (CFA) were conducted to examine the construct validity of the eight LLL determinants (mastery goals, performance approach goals, performance avoidance goals, interest, self-efficacy, surface strategies, deep strategies, meta-cognitive strategies) and classroom structure (task, autonomy and recognition loading on the second order factor classroom structure). As many variables of interest in educational research cannot be directly observed, latent variables are supposed to capture the essence, the common variance, of the different observed variables and represent the construct more validly. Goodness-of-fit of the models was evaluated using several different indices, including χ^2^ Test of Model Fit, the Tucker-Lewis Index (TLI), Comparative Fit Index (CFI), and root mean square error of approximation (RMSEA). In addition, a 90% confidence interval around the point estimate enabled an assessment of the precision of the RMSEA estimate (for details of these indices, see Kline, 2011). Although there are no golden rules on cut-off values for good model fit (Marsh et al., 2004), we used traditional cutoff scores indicative of excellent and adequate fit to the data, respectively: (a) CFI and TLI ≥0.95 and ≥0.90, and (b) RMSEA ≤0.06 and ≤0.08. We tested separate CFA models for each construct. An evaluation of model fit (CFI, TLI, RMSEA) indicated a good model fit for each of the eight tested models.

In a second step, structural equation modeling (Kline, 2011) was employed to investigate the associations between perceived classroom structure, LLL determinants (mastery goals, performance approach goals, performance avoidance goals, interest, self-efficacy, surface strategies, deep strategies, meta-cognitive strategies) and achievement (last grade in school report, achievement test result) in one comprehensive model. Achievement test percentile rank values were z-standardized using the error function prior to analyses. We provide standardized coefficients. Standardized coefficients represent the amount of change in the outcome that can be expected from a standard deviation unit change in the predictors. Following Cohen’s (1988) guidelines, in the context of regression parameters, standardized values greater than 0.10, 0.30, and 0.50 generally reflect small, moderate, and large effect sizes.

The influence of domain and sex was investigated in preliminary analyses using ANOVA. Due to the large sample size the use of effect sizes to check also for practical relevance is crucial. In the context of ANOVA $\eta_{p}^{2}$ with values greater than 0.01, 0.09, and 0.25 indicating small, moderate, and large effect sizes. Main effects of domain were found for selected variables but they had no (self-efficacy) or only small (surface strategies, deep strategies) practical relevance (all $\eta_{p}^{2}$ < 0.028). Main effects of sex were found for selected variables but they had no practical relevance (mastery goals, performance goals, interest) or only small practical relevance (surface strategies, deep strategies, meta-cognitive strategies; all $\eta_{p}^{2}$ < 0.042). All significant main effects with at least small effect sizes ($\eta_{p}^{2}$ > 0.009) were considered in the main analysis.

## Results

### LLL Competencies of Austrian Students

The average responses for motivational variables were in the middle to upper scoring regions, with the highest mean scores for self-efficacy and mastery goals, followed by interest, performance avoidance goals, and performance approach goals. The highest mean scores for learning strategies are reported for deep strategies, followed by surface strategies and meta-cognitive strategies. Descriptive statistics with regard to all items and scales of the LLL constructs can be found in **Table 1**.

**Table 1:** Descriptive statistics for all items and scales.

Variable	Item	*M*	*SD*	Minimum	Maximum	Median	Kurtosis	Skewness
Mastery goals	1	2.79	1.06	1	4	3	-1.10	-0.36
	2	2.42	1.03	1	4	2	-1.14	0.11
	3	2.27	1.04	1	4	2	-1.09	0.31
	4	2.67	1.06	1	4	3	-1.18	-0.24
	5	2.64	1.09	1	4	3	-1.28	-0.18
	6	2.71	1.03	1	4	3	-1.07	-0.28
	
	Scale	2.58	1.05	1	4	2.67	-0.88	-0.16

Performance approach goals	1	2.27	1.09	1	4	2	-1.23	0.27
	2	2.31	1.07	1	4	2	-1.23	0.19
	3	2.43	1.09	1	4	2	-1.30	0.04
	4	2.12	1.06	1	4	2	-1.00	0.51
	
	Scale	2.28	1.08	1	4	2.25	-1.32	0.20

Performance avoidance goals	1	2.37	1.10	1	4	2	-1.31	0.15
	2	2.26	1.11	1	4	2	-1.27	0.31
	3	2.20	1.11	1	4	2	-1.21	0.39
	4	2.47	1.11	1	4	3	-1.35	0.00
	
	Scale	2.32	1.11	1	4	2.25	-1.12	0.15

Interest	1	2.80	0.97	1	4	3	-0.87	-0.34
	2	1.90	1.02	1	4	2	-0.66	0.77
	3	2.40	1.03	1	4	2	-1.13	0.14
	
	Scale	2.37	1.01	1	4	2.33	-0.79	0.20

Self-efficacy	1	3.27	0.86	1	4	3	0.39	-1.06
	2	3.15	0.90	1	4	3	-0.26	-0.79
	3	3.23	0.89	1	4	3	0.02	-0.95
	
	Scale	3.22	0.88	1	4	3.33	0.18	-0.85

Surface strategies	1	2.81	1.02	1	4	3	-0.97	-0.41
	2	3.39	0.83	1	4	4	0.76	-1.24
	3	3.14	0.97	1	4	3	-0.34	-0.86
	
	Scale	3.11	0.94	1	4	3.33	0.09	-0.77

Deep strategies	1	3.08	0.94	1	4	3	-0.44	-0.73
	2	3.29	0.91	1	4	4	0.33	-1.14
	3	3.49	0.74	1	4	4	1.69	-1.45
	
	Scale	3.29	0.86	1	4	3.33	0.92	-1.00

Meta-cognitive strategies	1	2.86	1.00	1	4	3	-0.91	-0.44
	2	2.73	0.98	1	4	3	-0.85	-0.37
	3	2.67	1.01	1	4	3	-1.03	-0.25
	4	3.43	0.78	1	4	4	1.03	-1.28
	5	3.10	0.99	1	4	3	-0.43	-0.82
	
	Scale	2.96	0.95	1	4	3	-0.01	-0.61

### Classroom Structure, Students’ LLL Competences, and Achievement

**FIGURE 1:**
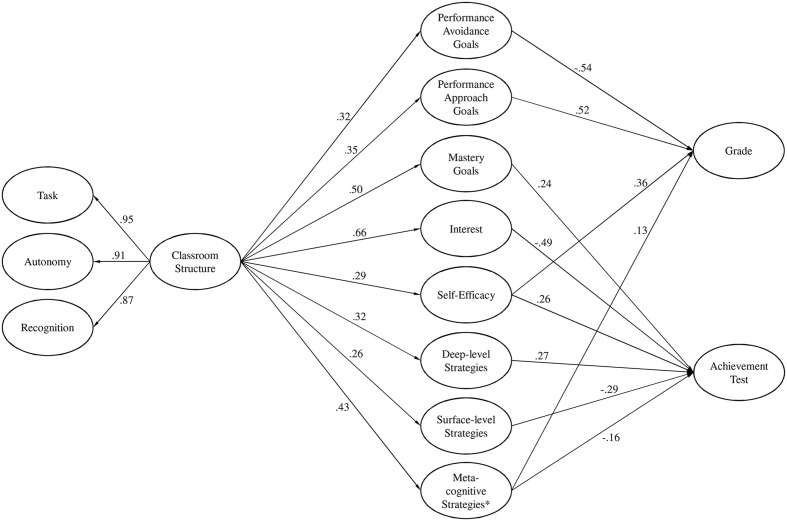
**Results concerning the structural model (classroom structure – lifelong learning determinants – achievement).** Standardized regression coefficient are reported and non-significant paths are not shown. Analyses were performed with Mplus 7.31; Achievement test percentile rank values were z-standardized using the error function prior to analyses. All other items were treated as ordered categorical, utilizing the WLSMV estimator. *Due to problems with multicollinearity meta-cognitive learning strategies were estimated in a separate model.

In order to investigate whether students’ perception of classroom structure is related to their LLL determinants (research question 2), and whether these LLL determinants are related to students achievement (research question 3), a structural equation model was fit to the data (see **Figure 1**). Preliminary analyses were conducted to control for possible multicollinearities between the LLL determinants that were used as predictors of achievement. The correlations between the latent variables deep-level strategies and meta-cognitive strategies were very high (>0.95). To avoid untrustworthy estimates and standard errors due to multicollinearities we conducted two models: one including all LLL determinants except meta-cognitive strategies and a separate model including only meta-cognitive strategies. For a better overview the results of both models were included in **Figure 1**.

Overall fit indices showed an excellent model fit for both the main model, χ^2^ (855, *N* = 5366) = 8326.199, *p* < 0.001, CFI = 0.968, TLI = 0.965, RMSEA = 0.040 [0.040, 0.041] and the separate meta-model, χ^2^ (204, *N* = 5366) = 3060.488, *p* < 0.001, CFI = 0.957, TLI = 0.952, RMSEA = 0.051 [0.049, 0.053]. Estimation revealed that classroom structure positively predicted students’ mastery goals (*b* = 0.50; *SE* = 0.01; *p* < 0.001), performance approach (*b* = 0.35; *SE* = 0.01; *p* < 0.001), performance avoidance (*b* = 0.32; *SE* = 0.01; *p* < 0.001), interest (*b* = 0.66; *SE* = 0.01; *p* < 0.001), self-efficacy (*b* = 0.29; *SE* = 0.02; *p* < 0.001), surface-level strategies (*b* = 0.26; *SE* = 0.02; *p* < 0.001), deep-level strategies (*b* = 0.32; *SE* = 0.02; *p* < 0.001), and meta-cognitive strategies (*b* = 0.43; *SE* = 0.02; *p* < 0.001). High perception of classroom structure predicts higher levels of LLL constructs. The largest coefficients were found for interest, mastery goals and meta-cognitive learning strategies.

Focusing on the LLL-achievement link estimation revealed that mastery goals (*b* = 0.24; *SE* = 0.05; *p* < 0.001), self-efficacy (*b* = 0.26; *SE* = 0.02; *p* < 0.001) and deep level strategies (*b* = 0.27; *SE* = 0.04; *p* < 0.001) positively predicted achievement test results, whereas interest (*b* = -0.49; *SE* = 0.06; *p* < 0.001), surface strategies (*b* = -0.29; *SE* = 0.04; *p* < 0.001), and meta-cognitive strategies (*b* = -0.16; *SE* = 0.01; *p* < 0.001) negatively predicted achievement test results. The largest coefficients were found for interest and surface-level strategies. No associations were found between performance approach goals (*b* = -0.04; *SE* = 0.15; *p* = 0.766), performance avoidance goals (*b* = -0.09; *SE* = 0.14; *p* = 0.544) and achievement test results.

Moreover, the grade in the last school report was positively predicted by performance approach goals (*b* = 0.52; *SE* = 0.14; *p* < 0.001), self-efficacy (*b* = 0.36; *SE* = 0.02; *p* < 0.001), and meta-cognitive strategies (*b* = 0.13; *SE* = 0.02; *p* < 0.001), indicating that high achievers show higher performance approach goal and self-efficacy expectations, and use more meta-cognitive learning strategies than low achievers. Performance avoidance goals (*b* = -0.54; *SE* = 0.13; *p* < 0.001) negatively predicted achievement test results. The largest coefficients were found for self-efficacy and performance approach goals. No associations were found between mastery goals (*b* = 0.08; *SE* = 0.05; *p* = 0.119), interest (*b* = -0.11; *SE* = 0.06; *p* = 0.056), surface-level (*b* = -0.22; *SE* = 0.03; *p* = 0.510) and deep-level strategies (*b* = 0.28; *SE* = 0.03; *p* = 0.510), and grade in last school report.

## Discussion

The present study aimed at investigating the LLL competencies of Austrian secondary school students. Additionally, classroom structure as a measure of teaching quality was tested as a predictor of their LLL competencies, and associations of students’ LLL competencies with their achievement were examined.

Concerning the first research question, descriptive results showed that the great majority of Austrian students – independent from domain and sex – do know and can apply self-regulated learning strategies as one component of LLL. Their values for surface and deep learning strategies as well as for metacognitive strategies were in the middle to upper scoring regions, meaning that the students think they apply those strategies rather often. Deep learning strategies, which are most commonly applied, have an impact on students’ performance on cognitive tests like the achievement test used in this study (Lukesch et al., 2002, 2004). However, metacognitive strategies, which are important with regard to grades in this study and usually show a positive, but modest relation with students’ achievement, with correlations ranging from 0.20 to 0.35 (Pintrich et al., 2000), are less common.

With regard to Austrian students’ motivation as the second component of LLL, the picture is less satisfactory: whilst students’ self-efficacy, which is strongly linked to grades in general (Zimmerman and Kitsantas, 2007) and also the most important for achievement in this study, is not the problem, there is a lack of interest in the school subjects. Furthermore, Austrian students’ quite often report to follow performance avoidance goals which are negatively linked to performance (Elliot and Murayama, 2008).

Compared to the results of large-scale assessment studies, there are some similarities, but also some differences in the results of our study. TIMSS and PISA generally show that students still have deficits with regard to lifelong learning competencies (Organisation for Economic Cooperation and Development [OECD], 2004). In our study, we found self-reported differences for some variables of LLL (metacognitive strategies and interest), but not for all. Concerning the evidence that key motivational determinants of LLL decrease after the transition to secondary school (Wigfield et al., 2006; Fischer and Rustemeyer, 2007; Schunk et al., 2008; Lüftenegger et al., 2012), we cannot confirm a decrease with our data since it is not longitudinal. However, the Austrian secondary students’ low values in interest in our study are in line with it and with the low level of interest reported in PISA 2006 (Schreiner, 2007). Both Austrian students in PISA 2003 (Organisation for Economic Cooperation and Development [OECD], 2007) and the Austrian secondary students in our study report high values of self-efficacy. Similarly, self-regulated learning values in our study correspond with the PISA 2000 results.

One special advantage of the present national study is the big sample size. Thus, even if these results are only of descriptive nature, they can give us a lot of insight into what to ameliorate in Austrian secondary education for promoting LLL. Klug et al. (2014) found in an interview study with Austrian teachers on how they try to foster their students LLL that the interviewed teachers indeed try to promote, for example, students’ interest. However, there seems to be little impact yet and much leeway left for improvement.

Since the questionnaire was administered in two very different domains, we could also gain insight into the nature of LLL in terms of domain-specificity. As we found nearly no practical relevant differences in LLL competencies between the major domains math and German, we assume that LLL could be considered as a generic competence.

With regard to the second research question, classroom structure positively predicted all of the LLL variables with regression coefficients in a range that argue for the high impact that the perception of their classroom instruction and teachers’ instructional practices has on students’ motivation and self-regulated learning (Kaplan and Maehr, 1999; Lüftenegger et al., 2012, 2014). Not only is the impact on students’ interest impressive, with 43% of the variance explained by the classroom structure, but also learning goal orientation, with 25% variance explained, as well as metacognitive strategies with 21% variance explained. This can obviously be effectively fostered by the kind of classroom structure the teacher creates.

These results are in turn relevant considering the findings for the third research question, where grades could be predicted positively by performance approach goals, self-efficacy and metacognitive strategies, and negatively by performance avoidance goals, which is in line with other research (e.g., Pintrich et al., 2000; Zimmerman and Kitsantas, 2007; Elliot and Murayama, 2008). The correlations between Austrian students’ LLL competencies and achievement are of a small to medium size and correspond with results from international monitoring studies (e.g., PISA, 2000) where the reported correlations of self-regulated learning and achievement are even a little lower than in this study (*r* = 0.12–0.18; see Svecnik and Reiter, 2002).

There are differences in the predictive power of the LLL variables for the two achievement measures we used. Grades are more strongly associated with motivational variables and meta-cognitive strategies than the achievement measures in our study, whereas only achievement test values are associated with cognitive learning strategies. An explanation could be that the achievement tests used in this study are cognitive tests which differ from what happens in the classroom. Thus the link between what happens in the classroom to promote students’ motivation and the results in the achievement tests is rather weak, whereas cognitive strategies are relevant for succeeding in these tests independently from what happens in the classroom. As Lüftenegger et al. (2015) point out, due to the lack of corresponding explicit feedback, students are less aware of their absolute performance levels and their relative standing on standardized tests than of their grades. Therefore, the association of achievement tests with motivational variables is limited, and school grades are likely to show a stronger link with motivation (Marsh et al., 2005). Interest, which can be most effectively promoted by the classroom structure provided by the teacher, even predicts achievement test scores negatively in this study, which again points to the achievement test being of little ecological validity. Meta-cognitive strategies are also a negative predictor of achievement test scores, but the regression coefficient is very small so that we see no practical relevance.

### Limitations of the Present Study

The most crucial limitation of the present study lies in its cross-sectional nature, which does not allow for causal interpretations of the regression analyses where LLL is predicted by classroom structure and achievement is predicted by LLL. The second limitation concerns the self-report character of the questionnaire (cp. Panadero et al., 2015), which is prone to biases like social desirability. One might guess that students wanted to present themselves as more motivated and self-regulated than they are in reality. That could explain the rather high values students indicated for the self-regulated learning strategies. Additionally students’ grades as an indicator for achievement were assessed retrospectively. Thus, current LLL competencies are correlated with former grades. Results would be more valid if we could measure both former and latter grades.

### Implications for Research and Practice

Austrian secondary school students’ LLL competencies are better developed than expected, at least in what students’ tell us in a self-report. However, there are parts of LLL, where we see need for action due to the results at hand: the low interest of Austrian students should be worked on in order to inspire students for learning. That is possible, especially by having the teacher create a motivating classroom structure. Furthermore, performance avoidance goal orientation should be lowered by actions in the classroom similarly, especially since the lower the performance avoidance goals, the better the grades.

In conclusion, the most impressive implication is that what the teacher does in terms of classroom structure is very important for students’ LLL as the really high correlations with interest, but also with self-efficacy, goal orientations and self-regulated learning strategies indicate. This is especially important if the correlations with grades are considered where goal orientations, self-efficacy, and metacognitive strategies matter.

Since LLL is considered and shown to be as important as promotable by creating a stimulant classroom structure, teachers should be enabled to create this kind of classroom structure. In order to do that, they need knowledge about the relevant dimensions of classroom structure and LLL, about how to promote them indirectly as well as directly, self-reflection about their own LLL and classroom management as well as supervised practice in promoting LLL. However, if you look at Austrian curricula for teacher education, promoting LLL has not been included for a long time, and in further education it is, at the utmost, one topic among many (Schober et al., 2009). Even after a recent revision in teacher education, there is only little space for dealing with related topics. Thus, we suggest to make the benefits of learning and practicing how to teach in a way that promotes LLL more evident for teacher education and further education.

## Author Contributions

All authors listed, have made substantial, direct and intellectual contribution to the work, and approved it for publication.

## Conflict of Interest Statement

The authors declare that the research was conducted in the absence of any commercial or financial relationships that could be construed as a potential conflict of interest.
